# Anxiety-Reducing Effects of Lavender Essential Oil Inhalation: A Systematic Review

**DOI:** 10.3390/healthcare11222978

**Published:** 2023-11-17

**Authors:** Onyoo Yoo, Sin-Ae Park

**Affiliations:** 1 Department of Bio and Healing Convergence, Graduate School, Konkuk University, Seoul 05029, Republic of Korea; onyoo@konkuk.ac.kr; 2 Department of Systems Biotechnology, Konkuk University, Seoul 05029, Republic of Korea

**Keywords:** anxiety, anxiolytic intervention, aromatherapy, inhalation, lavender essential oil

## Abstract

Anxiety disorders are the most prevalent and disabling mental disorders, causing health-related burdens. With the increasing demand for and interest in safe and acceptable anxiolytics, several studies report the anxiolytic effects of lavender aromatherapy, providing evidence of its physiological and psychological effects. However, existing reviews comprehensively cover the effects of different modes of delivering aromatherapy. Therefore, this review assesses the efficacy of lavender essential oil inhalation in reducing anxiety. The titles and abstracts of relevant articles published over the last five years were searched in PubMed, Web of Science, and Scopus databases. This review only included clinical trials that utilized lavender inhalation for anxiety treatment. Eleven studies comprising 972 participants were included. Of these, 10 reported significantly decreased anxiety levels after lavender oil inhalation. The physiological measures of vital signs, including blood pressure, heart rate, respiratory rate, pulse, and saturation, were conducted in three trials, showing that lavender oil inhalation could physiologically affect anxiety levels. Lavender oil inhalation is a safe and feasible anxiolytic intervention for treating people with diverse types of anxiety. Data from further studies with a high-quality design and accurate information are necessary to confirm the validity of these findings and elucidate the anxiety-reducing mechanisms of lavender inhalation.

## 1. Introduction

Anxiety disorders are the most prevalent mental disorders [1] and include panic disorder (with or without agoraphobia), separation anxiety disorder, specific phobias, social anxiety disorder, and generalized anxiety disorder [2]. Anxiety disorders are significantly comorbid with other psychiatric disorders, such as another anxiety disorder or major depression [3,4], making treatment more complex. Moreover, along with depressive disorders, anxiety disorders are the most disabling mental disorders, causing health-related burdens [5].

Anxiety is a negative emotional state caused by a dysfunction in the brain’s circuits that regulate emotional responses to potential threats [6]. The modulation of anxiety can be explained by increased excitatory neurotransmission through glutamate or decreased inhibitory neurotransmission through gamma-aminobutyric acid (GABA) [6,7]. These pathways play a significant role in the regulation of anxiety and are key targets for treating anxiety disorders [7]. Anxiety is also linked to a deficiency in monoamine neurotransmitters (dopamine, noradrenaline, and serotonin) and the dysregulation of neurotransmitter receptors [8].

The recommended initial treatment options for anxiety disorders are cognitive-behavioral therapy, pharmacotherapy, or a combination of the two [9]. Recent research is also utilizing neuroimaging, genetic, and blood-based approaches to identify pathogenetic and treatment-related biomarkers for anxiety disorders [10]. The first-line medications recommended for anxiety disorders are selective serotonin reuptake inhibitors (SSRIs) and selective serotonin-norepinephrine reuptake inhibitors (SNRIs) because of their beneficial effects and risk balance. Benzodiazepines are not recommended owing to their potential side effects [11]. SSRIs and SNRIs are effective treatments for anxiety disorders, with higher doses of SSRIs being associated in reports with a greater likelihood of treatment response and improved symptomatology [12]. However, it has also been found that higher doses are more likely to cause treatment discontinuation in patients due to side effects [12]. The chronic use of synthetic neuropsychiatric drugs can cause psychological and physical side effects, such as headaches, sleep problems, tiredness, anxiety, sexual dysfunction, weight gain, dry mouth, and elevated blood pressure [13]. Symptoms usually tend to recur when medications are discontinued, and relapses are common even when patients follow instructions for administering medication [14]. Patients with anxiety face concerns about the psychological burden of having to take medication continuously, as well as the potential side effects. Thus, the demand for safe and acceptable anti-anxiety drugs, especially in alternative medicine, is increasing.

Aromatherapy is a field of alternative medicine or complementary and alternative medicine [15,16,17]. It refers to the use of essential oils extracted from medicinal plants to help restore the balance of the body and mind. The term “aromatherapy” was first coined by the French chemist and perfumer Gattefosse in the book *Aromatherapie*, published in 1937 [16,18,19]. However, the practice of extracting and using aromatic oils from plants has existed globally in various forms since ancient times, long before the term “aromatherapy” was coined [20,21,22].

Essential oils, known as secondary metabolites, are biochemical aromatic substances produced by plants for survival in the environment; they are mainly diffused onto the plant surface through a special secretory tissue [22,23]. Essential oils can be extracted from various parts of the plant, such as flowers, leaves, seeds, fruit peels, heartwood, resins, and roots [15]. These organic compounds consist of numerous molecular structures of hydrocarbons and oxygenated derivatives, including terpenoids and phenylpropanoids that come from different biosynthetic pathways [22,24,25]. Extensive research has been conducted on essential oils because of their ability to pass through cell membranes and affect various molecular targets [26]. Their biological effects have been extensively researched, and their antioxidant, antimicrobial, antifungal, antiparasitic, anti-inflammatory, antinociceptive, and antitumor effects have been confirmed [22,26]. Essential oils have over 3000 constituents, of which approximately 300 are used commercially by the food, cosmetic, and pharmaceutical industries [27]. Aromatherapy, which utilizes essential oils for their therapeutic properties, is commonly used to treat both mental and physical health problems.

Essential oils are used in several delivery modes, such as inhalation, massage, bath, compression, and topical skin application [16]. Inhalation is the primary method used in aromatherapy because odors can affect mood, behavior, and physiology [28]. Through inhalation, essential oils influence the neurotransmission pathways that affect emotions [29] and stimulate the brain to release chemicals like serotonin and dopamine, which help regulate mood [30].

Lavender is one of the most commonly used essential oils in aromatherapy for various clinical purposes, including nervousness, insomnia, spasms, pain, headaches, depression, and anxiety [31,32,33]. Lavender belongs to the Lamiaceae family, and the most commonly grown species for producing essential oils is *Lavandula angustifolia*, also known as true lavender or English lavender [31].

The anxiolytic effects of lavender essential oils in patients with different conditions have been reported in several studies, which provide evidence for their physiological and psychological benefits [34,35,36]. However, studies reviewing the anxiolytic effects of lavender essential oils have not accounted, in detail, for the effects of different modes of delivering aromatherapy. Therefore, excluding other delivery modes, this study aims to systematically review the recent scientific literature on the efficacy of lavender inhalation for anxiety reduction in relevant clinical trials.

## 2. Materials and Methods

### 2.1. Data Sources and Search Strategy

A systematic review following the PRISMA guidelines was conducted to investigate the anxiety-reducing effect of inhaling lavender essential oil. Relevant studies on the anxiety-reducing effects of lavender fragrance published in the last five years were analyzed.

In April 2023, a keyword search-based review was conducted using Advanced Search on PubMed, Web of Science, and Scopus databases. Titles and abstracts were searched using the following search terms: (lavender OR lavandula) AND (inhalation OR inhale OR inhaled OR olfactory OR smell) AND (anxiety OR anxious OR anxiolytic).

### 2.2. Eligibility Criteria

This review was limited to English language papers published between 1 January 2018, and 31 December 2022. The inclusion criteria were as follows: (1) studies exploring the anxiety-reducing effect of inhaling lavender essential oil; (2) studies based on experiments with human participants; (3) studies following a treatment–control experimental approach; (4) studies that used self-rated or physiological indicators of anxiety.

This review focused on the anxiety-reducing effects of inhaled lavender oil; other effects of lavender and the effects of other delivery modes of aromatherapy were not included. Studies were excluded if they used a blend of lavender and other herbal essential oils or if lavender oil inhalation was not the main intervention. All lavender species were included. However, studies that did not clearly specify the species (scientific name) were excluded.

### 2.3. Study Selection and Data Extraction

Preliminary screening was conducted by reviewing titles and abstracts, followed by the full-text screening of the included articles using the predetermined eligibility criteria.

The following data were collected from the included articles: the first author’s name, publication year, study design, lavender species, the number and characteristics of participants, the number of analyzed patients/controls, intervention (a brief description of the intervention), the type of control, anxiety outcomes, significant intergroup differences, the author’s conclusion, and reported adverse events.

## 3. Results

### 3.1. Results of the Literature Search

**Figure 1:**
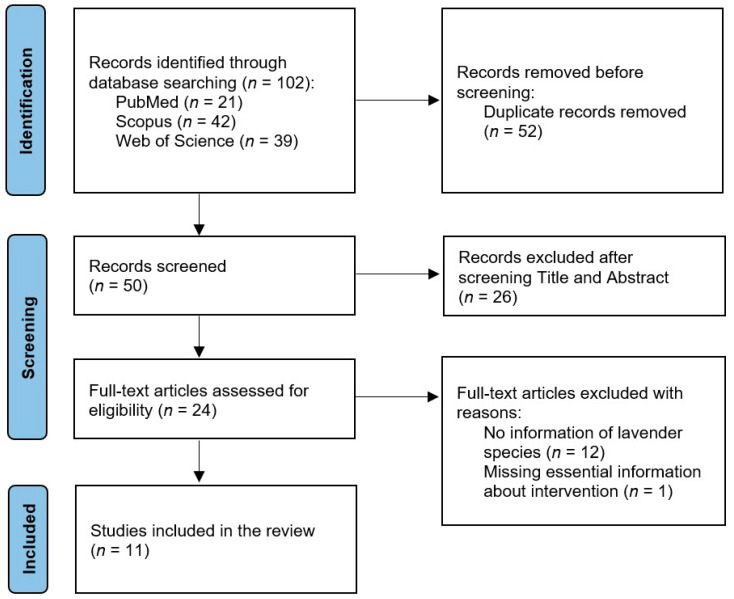
Flow diagram of the literature review and selection process.

A total of 102 papers were retrieved from the electronic database search. After removing 52 duplicates and screening titles and abstracts, 24 articles were retained for a full-text assessment. Only 11 studies met the eligibility criteria. Figure 1 shows the selection process.

### 3.2. Study Characteristics and Outcomes

In Table 1, we summarize the main data from the included studies. All the 11 included studies were randomized controlled trials. A total of 972 participants were included, 431 of whom underwent lavender aromatherapy intervention. The trial sample sizes ranged between 34 and 183 participants. Most participants were adults, except in one study, which involved 126 children [37]. Six studies adopted a two-arm parallel group design, and five adopted a three-arm parallel group design. These studies tested the anxiolytic efficacy of lavender essential oil in patients undergoing general [38], orthognathic [39], cataract [40], and benign prostate hyperplasia surgeries [41]. Furthermore, it was tested among patients with diagnosed depression undergoing electroconvulsive therapy [42], patients with a myofascial pain syndrome undergoing their first trigger-point injections [43], hemodialysis patients [44], women undergoing intrauterine insemination [45], postmenopausal women with depression [46], children awaiting tooth extraction [37], and older adults [47].

The most used lavender species in the included studies was *Lavandula angustifolia*. Among the 11 studies, eight studies used essential oil from *L. angustifolia* (the synonym *L. officinalis* was included as *L. angustifolia*), and the other three studies used *L. stoechas*, *L. latifolia*, and *L. × intermedia*, respectively. In most studies, two to six drops of lavender essential oil were used for inhalation via cotton balls, med patches, napkins, gauze pads, handkerchiefs, or diffusers, whereas one study reported the use of 20 drops through a vaporizer. Eight studies were designed for patients undergoing surgery or therapy—which are anxiety-inducing situations—and implemented aromatherapy once, whereas three studies conducted aromatherapy every night for one to four weeks among patients with anxiety in their daily lives. Participants in the control groups received placebos, routine care, or no intervention. Placebos mostly use water (distilled water and water vapor) or oil (baby oil and grapeseed oil) without a fragrance.

Spielberger’s State–Trait Anxiety Inventory was the most frequently used tool to evaluate anxiety across the included studies. Six of the eleven studies included in this review used the Spielberger State–Trait Anxiety Inventory to measure anxiety. The Face Image Scale, Depression Anxiety Stress Scale, Hospital Anxiety and Depression Scale, Visual Analogue Scale, Beck Anxiety Inventory, and Hamilton Anxiety Assessment Scale were used either alone or in combination with other scales. Three studies used vital signs to estimate anxiety levels indirectly, together with other anxiety scales.

### 3.3. Efficacy of Lavender Oil Inhalation as an Anxiolytic Therapy

Anxiety level evaluation scales were measured in all 11 trials, and 10 studies reported significantly decreased anxiety with lavender oil inhalation. The statistical significance of the reported studies ranged from *p* < 0.001 to *p* < 0.05. Compared with the control group, the lavender intervention group of children before tooth extraction experienced a significant decrease in anxiety levels (*p* < 0.023) [37]. Significant decreases in anxiety levels were observed in adult patients undergoing general surgery (*p* < 0.001) [38], elderly patients undergoing benign prostate hyperplasia surgery (*p* < 0.05) [41], women undergoing intrauterine insemination (*p* < 0.02) [45], adult patients with a myofascial pain syndrome receiving trigger point injections (*p* < 0.001) [43], adult patients undergoing electroconvulsive therapy (*p* < 0.001) [42], and adult patients awaiting cataract surgery (*p* < 0.023) [40]. The groups that received the long-term lavender intervention also demonstrated a notable reduction in their anxiety levels. Further, lavender helped decrease anxiety in older adults who underwent a 30-night intervention (*p* < 0.01) [47], postmenopausal women with depression who received treatment for four weeks (*p* < 0.001) [46], and adult patients undergoing hemodialysis with sleep problems and anxiety who were treated for one week (*p* < 0.001) [44]. However, one trial involving adult patients undergoing orthognathic surgery reported no significant anxiolytic effects in regard to psychological anxiety [39].

The physiological measurements of vital signs—including blood pressure, heart rate, respiratory rate, pulse, and saturation—were conducted in three trials. In one trial involving children undergoing tooth extraction, the vital signs of the lavender group were significantly lower than those of the control group (*p* < 0.05), except for saturation levels [37]. Another trial involving elderly patients undergoing benign prostate hyperplasia surgery reported significant differences in their respiratory rates and saturation of percutaneous oxygen (SPO2) (*p* < 0.05), whereas no significant differences were reported in blood pressure and pulse [41]. A trial of elderly patients awaiting cataract surgery reported no significant differences in their vital signs compared with the control group. Nevertheless, significant changes were reported intragroup for systolic blood pressure (*p* = 0.03), pulse (*p* < 0.001), and respiration (*p* < 0.001) [40]. The results of the vital sign measurements showed that lavender oil inhalation can physiologically affect anxiety levels.

Among the 11 studies, 10 reported that the inhalation of lavender essential oil decreased anxiety. However, one study reported that lavender essential oil diffusion did not have an anxiolytic effect on patients undergoing orthognathic surgery [39]. It is worth noting that studies reported that exposure to lavender aromatherapy had a positive impact on anxiety reduction, regardless of the indicator or lavender species used. Overall, the results showed a strong positive correlation between the inhalation of lavender essential oil and its anxiolytic effects.

## 4. Discussion

In this study, the effects of lavender inhalation on anxiety were reviewed based on evidence published between 1 January 2018 and 31 December 2022. This review included 11 trials, which showed that lavender inhalation had a significant anxiety-reducing effect on the psychological and physiological manifestations of anxiety.

Lavender includes several species with different chemical characteristics. The four main species are *Lavandula angustifolia*, *Lavandula latifolia*, *Lavandula stoechas*, and *Lavandula × intermedia.* These species have differences in aroma and reported therapeutic uses [32]. The main constituents of lavender essential oil are linalool, linalyl acetate, 1,8-cineole, ß-ocimene, terpinen-4-ol, and camphor [32,48]. The key active constituents are linalyl acetate and linalool, which are responsible for the calming and sedative effects of lavender and are also the major constituents of *L. angustifolia* [49].

This review focused on inhalation as a delivery mode for aromatherapy interventions. Previous review studies suggest that inhalation is the most effective and feasible option for the short-term treatment of anxiety compared to other modalities [34,35]. Another review reported that inhalation administration is easy, inexpensive, safe, and noninvasive [36]. Essential oils, when inhaled, have an effect on both the olfactory and respiratory systems [50]. The shortest connection between the central nervous system and the external environment is the olfactory nerve [51]. Odor molecules interact with olfactory receptor neurons, generating unique electric signals that are sent to the brain and have a regulatory effect on mood [50]. In summary, essential oils interact with central nervous system receptors, leading to physiological changes that can have psychological effects [52].

Previous studies have shown that lavender essential oils have anxiolytic effects. These effects can be attributed to an increase in serotonin levels [53], where the action on gamma–aminobutyric acid receptors as linalool produces an effect through interactions with the GABAergic pathway [54] or the induction of a sedative effect by interacting with the hypothalamic–pituitary–adrenal axis to lower cortisol levels in the serum [52]. The anxiolytic effects of lavender oil are also associated with the antioxidant regulation of H2O2 production by ascorbate [29]. Another study found that inhaling lavender oil significantly increased the alpha power and low beta power spectrum, indicating the resting and stress-free state of the brain [55].

The present review has a limitation in that while many studies reported the anxiety-reducing effects of lavender inhalation, they did not provide essential information on the lavender species used in the trial. Twelve additional studies that explored the anxiolytic effect of lavender inhalation over the last five years were excluded from this review as they did not specify the plant species (scientific names) used in their trials [56,57,58,59,60,61,62,63,64,65,66,67]. The chemical composition of essential oils depends on the plant species and its variety, the part collected, its origin, climate, soil, the agrochemicals used, stocking time, preparation, the method of extraction, and other factors [68]. The therapeutic effects of essential oils mainly depend on their active compounds, and the combination of diverse chemical compounds in essential oils may interact with different neurotransmitter pathways, resulting in numerous therapeutic effects [50]. Accordingly, studies performed with essential oils should always specify the scientific name of the plant used, which should be considered essential information to ensure the quality and accuracy of data and the safety of research. Despite the importance of providing information on the lavender species used, many studies did not, which resulted in an inability to identify the scientific name of the lavender.

Other possible limitations include the absence of clinical participants, such as patients with anxiety disorders or subclinical participants exhibiting elevated levels of anxiety. The studies primarily focus on stressful situations within medical contexts. It is desirable to have more appropriate measures for the symptoms of anxiety disorders. Despite our best efforts to cover all possible studies, it is still conceivable that some research may have been overlooked, as our search was limited to English language papers. The results of this review should be considered with caution owing to these limitations.

## 5. Conclusions

The results of this review showed decreased anxiety levels regardless of the lavender species used. This review provides evidence supporting the anxiety-reducing efficacy of lavender essential oil inhalation. Inhaling lavender essential oil appears to be an effective, safe, and feasible treatment option for anxiety. Based on the current evidence, the inhalation of lavender essential oil can be recommended as an efficacious anxiolytic solution to improve coping in people facing diverse anxiety situations. Furthermore, the results of this review can be referred to for designing psychological interventions in participants with anxiety disorders who expect positive emotional effects.

However, the conclusions of the review are provisional in nature. Data from further random controlled trials with a high-quality design and accurate information are necessary to confirm the validity of these findings and elucidate the anxiety-reducing mechanisms of lavender oil inhalation. In addition, the amount and method of lavender oil inhalation utilized in the reviewed studies varied widely. In future studies, it is crucial to define the qualifications of oils that can be used therapeutically, establish guidelines for dosages, and gather accurate information on safe inhalation methods.

## Figures and Tables

**Table 1:** Characteristics of the 11 reviewed studies.

Author, Year	Study Design	Lavender Species	(No. of Participants) Participants Characteristics	Intervention Group	Control Group	Anxiety Evaluation	Significant Difference Intergroup	Authors’ Conclusion	Adverse Events
Arslan et al., 2020 [37]	RCT	*Lavandula* *angustifoila*	(126) Children aged 6–12 years assigned to undergo tooth extraction	(63) Two drops of lavender oil on med patches; patients inhaled the oil without skin contact for 3 min	(63) No prior application	FIS, Vital signs (BP, HR, pulse, saturation)	Yes (*p* = 0.023) Vital signs (except saturation), Yes (*p* < 0.05)	‘Lavender oil can be preferred as a treatment of choice in routine pediatric dentistry.’	None
Bozkurt and Vural, 2019 [39]	RCT	*Lavandula* *angustifoila*	(90) Adult patients aged 18–45 years scheduled to undergo orthognathic surgery	(30) 0.1 mL of lavender oil diffusions in 120 mL of water during a 1 h period before surgery (30) The same procedure, but 0.3 mL of lavender oil was used	(30) The same procedure as that in the intervention group, but water diffusion was performed without oil	STAI	No	‘1 h of presurgical inhalation of 0.1 mL and 0.3 mL lavender oil diffusions in 120 mL of water did not have an anxiolytic effect on patients undergoing orthognathic surgery…’	NA
Ebrahimi et al., 2022 [47]	RCT	*Lavandula* *stoechas*	(183) Older adults at least 65 years of age	(61) 3 drops of lavender EO on a cotton ball pinned to the pillow cover every night for 30 nights (61) The same procedure, but chamomile EO was used	(61) The same procedure as that in the intervention group, but distilled water was used	DASS	Yes (*p* < 0.01)	‘Inhalation aromatherapy with both lavender and chamomile EO helped decrease depression, anxiety, and stress levels in community-dwelling older adults.’	NA
Ebrahimi et al., 2021 [38]	RCT	*Lavandula* *angustifoila*	(90) Adult patients over 40 years old undergoing general surgery	(30) 2 drops of lavender EO on a napkin to inhale for 20 min at a distance of 20 cm, 60 min before the start of surgery (30) The same procedure, but *Citrus aurantium* EO was used	(30) The same procedure as that in the intervention group, but distilled water without a fragrance was used	STAI	Yes (*p* < 0.001)	‘Inhalation of *Citrus aurantium* and lavender aroma reduces anxiety in male and female patients.’	None
Genc and Saritas, 2020 [41]	RCT	*Lavandula* *× intermedia*	(110) Elderly male patients (age range unspecified) hospitalized at the urology clinic scheduled to undergo benign prostate hyperplasia surgery	(55) 5 drops of lavender EO on a sterile gauze or a cotton ball was inhaled for 5 min kept at 7–10 cm from the nose	(55) Standard nursing care in compliance with the routine clinical procedure	STAI, Vital signs (BP, RR, pulse, SPO2)	STAI, Yes (*p* < 0.05) RR, SPO2, Yes (*p* < 0.05) BP, Pulse, No	‘Lavender oil inhalation reduced anxiety levels and had effects on the vital signs of benign prostate hyperplasia patients in their preoperative period.’	NA
Jokar et al., 2020 [46]	RCT	*Lavandula* *latifolia*	(46) Postmenopausal women aged above 45 years with a depression score greater than 10 on the Beck Depression Inventory	(23) 2 drops of lavender EO on a handkerchief was attached to a collar and removed after 20 min every night before sleep for 4 weeks	(23) The same procedure as that in the intervention group, but distilled water was used	STAI	State anxiety, Yes (*p* < 0.001) Trait anxiety, No	‘Lavender aromatherapy may be an effective noninvasive treatment during the postmenopausal stage.’	None
Jones et al., 2021 [45]	RCT	*Lavandula* *angustifoila*	(62) Women aged 18–45 years undergoing intrauterine insemination at a hospital-based fertility clinic	(31) Patients smelt a pouch containing a cotton ball with 1 drop of lavender EO during the procedure	(31) The same procedure as that in the intervention group, but a cotton ball with 1 drop of water was used	HADS, VAS	Yes (*p* = 0.02)	‘Lavender aromatherapy reduced anxiety and was preferred by women during intrauterine insemination fertility treatments.’	None
Kasar et al., 2020 [43]	RCT	*Lavandula* *angustifoila*	(66) Adult patients (age range unspecified) of a myofascial pain syndrome undergoing trigger point injections for the first time	(22) 5 drops of lavender oil were dispersed through a diffuser (100 cc distilled water) placed 30 cm away from the participants during the trigger point process	(22) The same procedure as that in the intervention group, but odorless baby oil was used (22) No intervention was made	STAI	Yes (*p* < 0.001)	‘Lavender oil inhalation was found to reduce pain and anxiety during trigger point injection and improve patient comfort…’	NA
Moghadam et al., 2022 [42]	RCT	*Lavandula* *angustifoila*	(90) Adult patients aged 18–60 years with a confirmed diagnosis of depression undergoing electroconvulsive therapy	(30) 2 drops of lavender EO on a sterile gauze pad was inhaled for 3–5 min before electroconvulsive therapy (30) Breathing exercises were performed before electroconvulsive therapy	(30) Routine care	BAI	Yes (*p* < 0.001)	‘Inhaled lavender EO and breathing exercises can be considered by clinical nurses as simple, applicable, and effective interventions to reduce electroconvulsive therapy-related anxiety in depressed patients.’	None
Sentürk and Kartın, 2018 [44]	RCT	*Lavandula* *angustifoila*	(34) Adults older than 18 years, including patients of hemodialysis with sleep problems and anxiety	(17) 2 drops of lavender oil on a cotton ball in a box placed 15–20 cm away from the pillow 30 min before going to bed for 1 week	(17) No intervention	HAM-A	Yes (*p* < 0.001)	‘Lavender oil inhalation can be easily applied by nurses to individuals experiencing hemodialysis-related anxiety and sleep problems.’	None
Stanley et al., 2020 [40]	RCT	*Lavandula* *officinalis*	(75) Adult patients aged 21–75 years awaiting cataract surgery	(39) 20 drops of lavender EO were placed in the vaporizer near the patient’s chair in the lounge, who breathed normally for 20 min	(36) The same procedure as that in the intervention group, but a new vaporizer with 20 drops of grape seed oil was used	STAI Vital signs (BP, RR, pulse)	STAI, Yes (*p* = 0.023) Vital signs, No	‘Lavender aromatherapy reduced anxiety in preoperative cataract surgery patients.’	None

Abbreviations: BAI, Beck Anxiety Inventory; BP, blood pressure; DASS, Depression, Anxiety, and Stress Scale; EO, essential oil; FIS, Face Image Scale; HADS, Hospital Anxiety and Depression Scale; HAM-A, Hamilton Anxiety Assessment Scale; HR, heart rate; NA, not assessed; RCT, randomized controlled trial; RR, respiratory rate; SPO2, saturation of percutaneous oxygen; STAI, Spielberger State-Trait Anxiety Inventory; VAS, Visual Analogue Scale.

## Data Availability

The data that support the findings of this study are available from the corresponding author upon reasonable request.
